# Sarcopenia in Hemodialysis Patients: Prevalence, Independent Risk Factors, and Functional Implications—A Multicenter Cross-Sectional Study

**DOI:** 10.3390/jcm14196893

**Published:** 2025-09-29

**Authors:** Rym Ben Othman, Amani Balti, Sabrine Boukhris, Halil İbrahim Ceylan, Henda Jamoussi, Raul Ioan Muntean, Ismail Dergaa

**Affiliations:** 1 Department A, Institut National Zouhair Kallel de Nutrition et de Technologie Alimentaire, 11 rue Jbel Lakhdar, Bab Saadoun, Tunis 1007, Tunisia; benothmanr@gmail.com (R.B.O.); hendajamoussi@gmail.com (H.J.); 2 Faculty of Medicine of Tunis, University of Tunis El Manar, 15 rue Djebel Lakhdhar, La Rabta, Tunis 1007, Tunisia; baltiamani098@gmail.com (A.B.); sabrineboukhris@gmail.com (S.B.); 3 Etiopathogenesis, Pathophysiology and Treatment Research Unit of Obesity (UR18ES01), Faculty of Medicine of Tunis, University of Tunis El Manar, Tunis 1007, Tunisia; 4 Physical Education and Sports Teaching Department, Faculty of Sports Sciences, Atatürk University, Erzurum 25240, Türkiye; 5 Department of Physical Education and Sport, Faculty of Law and Social Sciences, University “1 Decembrie 1918” of Alba Iulia, 510009 Alba Iulia, Romania; 6 High Institute of Sport and Physical Education of Ksar Said, University of Manouba, Manouba 2010, Tunisia; phd.dergaa@gmail.com; 7 Physical Activity, Sport and Health Research Unit (UR18JS01), National Observatory of Sports, Tunis 1003, Tunisia

**Keywords:** chronic kidney disease, diabetes mellitus, muscle mass, physical performance, protein-energy wasting, quality of life, Barthel index, sarcopenia, hemodialysis

## Abstract

**Background:** Sarcopenia is a critical complication in hemodialysis patients, associated with poor clinical outcomes, increased morbidity, and reduced quality of life. Despite this, its significance, prevalence, and risk factor data in developing countries remain limited. **Objective:** This study aimed to determine the prevalence of sarcopenia and identify its independent risk factors in patients undergoing maintenance hemodialysis, while evaluating its impact on physical performance, nutritional intake, and quality of life. **Methods:** A multicenter cross-sectional study was conducted across three hemodialysis units in Tunisia. Sarcopenia was diagnosed using EWGSOP2 (European Working Group on Sarcopenia in Older People 2) criteria based on muscle strength, muscle mass, and physical performance. Handgrip dynamometry, mid-arm and calf circumferences, gait speed, Short Physical Performance Battery (SPPB), and Timed Up and Go (TUG) test were employed. Nutritional intake was assessed using a 7-day food history. Quality of life and functional status were evaluated using the SF-36 and Barthel Index, respectively. Logistic regression was used to identify independent predictors of sarcopenia. **Results**: Among 118 patients (mean age 56.74 ± 14.44 years), the prevalence of sarcopenia was 42.4% (*n* = 50). Sarcopenic individuals exhibited significantly poorer physical performance than their non-sarcopenic counterparts. Marked reductions were observed in handgrip strength (*p* < 0.001, *d* = −1.60, very large), SPPB scores (*p* < 0.001, *d* = −1.55, very large), and increased TUG time (*p* < 0.001, *d* = 1.46, very large), indicating substantial functional impairment. Limb circumferences were also significantly lower in the sarcopenic group, including calf circumference (*p* = 0.002, *d* = −1.39, large) and mid-arm circumference (*p* = 0.013, *d* = −0.87, large). Gait speed was slower (*p* = 0.010, *d* = −0.40, small to moderate). Health-related quality of life was significantly compromised in sarcopenic individuals, with lower SF-36 total scores (*p* = 0.001, *d* = −1.96, very large) and reduced functional independence as measured by the Barthel Index (*p* = 0.010, *d* = −0.97, large). Hemoglobin levels were also significantly lower in the sarcopenic group (*p* = 0.048, *d* = −0.96, large). Dietary assessment revealed lower fiber intake (*p* = 0.006, *d* = 1.80, very large) and reduced magnesium consumption (*p* = 0.020, *d* = 0.94, large) among individuals with sarcopenia. In the multivariate logistic regression analysis, diabetes mellitus (OR = 2.14, 95% CI: 1.30–3.67, *p* < 0.001) and longer duration of hemodialysis (OR = 1.56, 95% CI: 1.20–2.71, *p* = 0.028) were identified as independent predictors of sarcopenia. A lower SPPB score (OR = 0.48, 95% CI: 0.35–0.65, *p* < 0.001) was associated with sarcopenia. **Conclusion:** Sarcopenia is highly common among hemodialysis patients and is independently linked to diabetes, treatment duration, and reduced physical performance. It significantly affects the quality of life and ability to perform daily activities. Routine screening with simple functional tests is crucial, especially in high-risk patients. Early intervention should include physical rehabilitation, nutritional support, and strict blood sugar management to decrease sarcopenia-related complications.

## 1. Introduction

End-stage chronic kidney disease affects approximately 3.9 million individuals globally, with 69% requiring hemodialysis for survival [1]. The hemodialysis population demonstrates exceptionally high mortality rates, with annual death rates exceeding 15–20%, substantially higher than age-matched general populations [1]. Beyond mortality concerns, hemodialysis patients experience progressive functional decline, reduced physical performance, and deteriorating nutritional status, collectively contributing to poor clinical outcomes [2].

Sarcopenia, a geriatric syndrome characterized by progressive loss of skeletal muscle mass, strength, and physical performance, has emerged as a critical complication in the hemodialysis population [3]. Described initially by Rosenberg in 1989 as age-related muscle mass decline [4], the understanding of sarcopenia has evolved significantly over the past three decades. The European Working Group on Sarcopenia in Older People (EWGSOP) initially defined sarcopenia based solely on muscle mass reduction. Still, subsequent revisions have emphasized the importance of muscle function, leading to the current EWGSOP2 criteria, which prioritize muscle strength as the primary diagnostic parameter [3]. Contemporary definitions distinguish between primary sarcopenia, which is related to the aging process, and secondary sarcopenia, associated with chronic diseases, including chronic kidney disease [5,6].

The pathophysiology of sarcopenia in hemodialysis patients involves complex, interconnected mechanisms that extend beyond normal aging processes [7]. Chronic kidney disease induces a state of chronic inflammation characterized by elevated levels of pro-inflammatory cytokines, including tumor necrosis factor-alpha and interleukin-6 [8]. These inflammatory mediators activate muscle protein degradation pathways while simultaneously suppressing protein synthesis [9]. Uremic toxins accumulate in patients with end-stage renal disease, directly impairing muscle function by disrupting cellular metabolism and promoting oxidative stress [10]. Additionally, protein-energy wasting syndrome, affecting a significant proportion of dialysis patients, creates a catabolic environment that promotes muscle loss [11].

Despite the clinical significance of sarcopenia in hemodialysis populations, several critical research gaps persist in the current literature. First, prevalence estimates vary dramatically across studies, ranging from 14% to 73%, mainly due to inconsistent diagnostic criteria and methodological differences [11]. Most studies have been conducted in developed countries, leaving substantial knowledge gaps regarding the prevalence of sarcopenia in developing nations, where healthcare resources may be limited. Second, while multiple risk factors have been proposed, few studies have employed robust multivariate analyses to identify independent risk factors while controlling for confounding variables [2]. Third, the relationship between sarcopenia and quality of life in hemodialysis patients remains incompletely characterized [12]. Fourth, dietary factors and their association with sarcopenia development in hemodialysis patients have received limited attention [13]. Finally, there is a lack of consensus on sarcopenia definitions in dialysis populations and a limited availability of longitudinal studies [14,15].

Based on these research gaps, this study aimed to determine the prevalence of sarcopenia among Tunisian hemodialysis patients using standardized EWGSOP2 criteria, identify independent risk factors associated with the development of sarcopenia, evaluate the impact of sarcopenia on quality of life, and examine the relationship between sarcopenia and functional independence in this vulnerable population.

## 2. Materials and Methods

### 2.1. Ethical Approval

The study was conducted in accordance with the Declaration of Helsinki guidelines and was approved by the Ethics Committee of the Tunisian National Institute of Nutrition and Food Technology (approval number: 01/2023). All participants provided written informed consent before study inclusion, with full disclosure of study objectives, procedures, and withdrawal rights.

### 2.2. Study Design

This cross-sectional, multicenter observational study was conducted across three hemodialysis facilities in Tunisia: the Mahmoud El Matri Hospital Hemodialysis Unit (Ariana), the El Manzah Private Hemodialysis Center, and the El Omrane Polyclinic. Data collection took place over six months (June 2023–November 2023).

### 2.3. Sample Size Calculation

Sample size determination utilized the Cochrane formula *n* = z^2^ × *p*(1 − *p*)/i^2^, where z represents the confidence level according to standard normal distribution (z = 1.96 for 95% confidence), *p* indicates estimated sarcopenia prevalence in hemodialysis patients (55% based on British data [16], and i denotes precision (5%) [17]. Based on previous studies reporting a sarcopenia prevalence of 55% in hemodialysis patients [16], the calculated sample size was *n* = 105.

### 2.4. Participants

**Figure 1:**
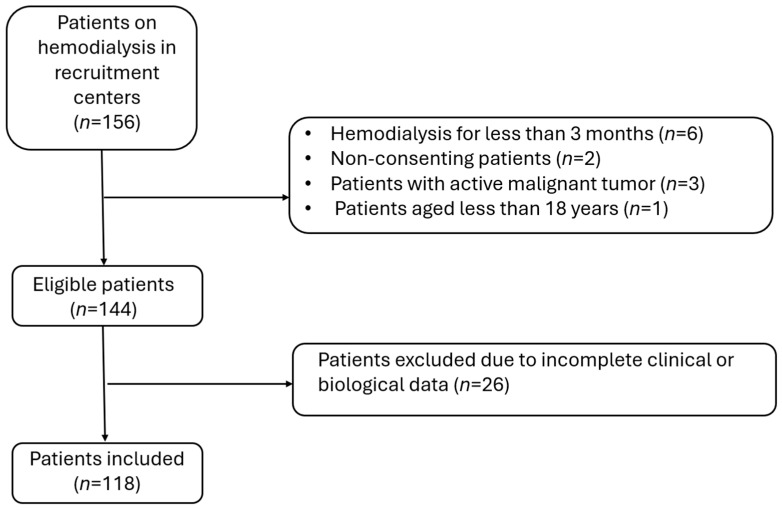
Flowchart of the population (*n* = 118).

Of the 156 patients undergoing maintenance hemodialysis initially screened across the participating centers, 12 were excluded due to the following reasons: duration of hemodialysis less than three months (*n* = 6), non-consent to participate (*n* = 2), active malignancy (*n* = 3), and age below 18 years (*n* = 1). This yielded a total of 144 eligible patients. An additional 26 patients were excluded due to incomplete clinical or biological data, resulting in a final analytical sample of 118 patients. Figure 1 illustrates the patient recruitment and selection process. Inclusion criteria comprised: regular hemodialysis treatment for ≥3 months, age ≥ 18 years, and provision of informed consent. Exclusion criteria included: inability to perform handgrip testing, presence of sarcopenia-related pathologies (cancer, myopathy, myositis), and cognitive impairment preventing questionnaire completion.

### 2.5. Experimental Procedures

Since this study is based on questionnaires, we ensured the highest standards in applying psychometric methods throughout the entire study protocol, as highlighted by Guelmemi et al. (2023) [18].

#### 2.5.1. Anthropometric Measurements

Height and weight were measured using standardized protocols. Body mass index (BMI) was calculated as weight (kg)/height (m^2^). Mid-arm circumference was measured at the acromion-olecranon midpoint using a tape measure on the non-fistula arm, with values < 22 cm indicating decreased muscle mass [19]. Calf circumference was measured at the maximum circumference in the supine position, with values < 31 cm reflecting muscle mass reduction [20].

#### 2.5.2. Muscle Strength Assessment

Handgrip strength was measured using a Camry EH101 dynamometer (Camry Scale, Zhongshan, China). Participants were seated with their backs supported, feet flat on the floor, and elbows flexed at 90°. Three trials were performed bilaterally, with maximum values recorded. Muscle weakness was defined as <27 kg for men and <16 kg for women according to EWGSOP2 criteria [3].

#### 2.5.3. Physical Performance Testing

The Short Physical Performance Battery (SPPB) evaluated balance, gait speed, and chair stand performance, with scores ≤ 8 indicating severe sarcopenia [21]. Gait speed was measured over 4 m, with values < 0.8 m/s indicating severe sarcopenia [22]. The Timed Up and Go (TUG) test required participants to rise from a chair, walk 3 m, turn, return, and sit, with completion times greater than 20 s indicating severe sarcopenia [23].

#### 2.5.4. Sarcopenia Screening

The SARC-F questionnaire assessed five domains: strength, assistance with walking, rising from a chair, climbing stairs, and a history of falls. Scores ≥ 4 indicated probable sarcopenia [24]. Sarcopenia diagnosis followed EWGSOP2 criteria, requiring evidence of low muscle strength plus either low muscle mass or low physical performance [3].

#### 2.5.5. Quality of Life Assessment

The SF-36 questionnaire evaluated eight health domains: physical functioning, role-physical, bodily pain, general health, vitality, social functioning, role-emotional, and mental health. Scores < 50 indicated impaired quality of life [25]. The Barthel Index assessed functional independence in activities of daily living, with scores ranging from 0 to 100 [26].

#### 2.5.6. Laboratory Analyses

Blood samples were collected pre-dialysis and analyzed in the central laboratory. Serum calcium was measured using photometric methods on a Cobas c311 analyzer (Roche Diagnostics, Mannheim, Germany). Hemoglobin levels were determined via spectrophotometry using a Sysmex XN-1000 hematology analyzer (Sysmex Corporation, Kobe, Japan). Triglycerides were assessed using enzymatic colorimetric methods on a Beckman Coulter AU480 (Beckman Coulter Inc., Brea, CA, USA). Serum albumin was measured with bromocresol green dye-binding techniques on the Cobas c501 platform (Roche Diagnostics, Mannheim, Germany). C-reactive protein (CRP) levels were determined using a high-sensitivity immunoturbidimetric assay on the Roche Cobas c502. Ferritin concentrations were quantified by enzyme-linked immunosorbent assay (ELISA) using the Siemens ADVIA Centaur XP (Siemens Healthineers, Tarrytown, NY, USA).

#### 2.5.7. Nutritional Assessment

Dietary intake was evaluated using the seven-day food history methodology. Patients received training before data collection, and a qualified nutritionist analyzed the results. This method has been previously validated and applied in dialysis patients, demonstrating acceptable accuracy in estimating dietary intake. Nutritional analysis was performed using NUTRILOG software, version 3.20 (Nutrilog SAS, Marans, France) to quantify energy, macronutrients, micronutrients, and fiber intake [27,28].

### 2.6. Statistical Analysis

Statistical analysis was conducted using SPSS software version 28.0 (IBM Corp., Armonk, NY, USA). The Kolmogorov–Smirnov test was used to assess the normality of the data distribution. Descriptive statistics included frequencies and percentages for categorical variables, as well as means ± standard deviations for continuous variables. Between-group comparisons utilized Student’s *t*-test or Mann–Whitney U test for continuous variables and chi-square or Fisher’s exact test for categorical variables. To quantify the magnitude of differences between sarcopenic and non-sarcopenic groups, effect sizes were calculated. For continuous variables, Cohen’s d was used, where values of 0.2, 0.5, 0.8, and ≥1.2 were interpreted as small, moderate, large, and very large effects, respectively. For categorical variables, Cramer’s V was applied, with thresholds of 0.1 (small), 0.3 (moderate), and 0.5 (large) for 2 × 2 contingency tables. These benchmarks provide a practical framework for evaluating the clinical significance of statistical differences beyond *p*-values [29,30]. Univariate analysis calculated odds ratios (OR) with 95% confidence intervals. Multivariate logistic regression identified independent risk factors, with statistical significance set at *p* < 0.05.

## 3. Results

### 3.1. Population Characteristics

A total of 118 patients were recruited (Figure 1), with 50 patients (42.4%) diagnosed with sarcopenia according to EWGSOP2 criteria (95% CI 34–51). All sarcopenic patients demonstrated reduced muscle strength, while reductions in muscle mass were identified in 30.5% of patients by calf circumference criteria and in 17.7% by mid-arm circumference criteria. Population characteristics are summarized in Table 1.

In Table 1, significant differences were observed between sarcopenic and non-sarcopenic patients across demographic, clinical, and functional parameters. Sarcopenic patients were significantly older than non-sarcopenic individuals (65.31 ± 14.19 vs. 56.15 ± 14.70 years, *p* < 0.001, d = 0.63, moderate) and had a longer duration of hemodialysis (148.47 ± 115.20 vs. 89.53 ± 98.70 months, *p* = 0.001, d = 0.56, moderate). Female sex was less prevalent in the sarcopenic group (32.0% vs. 55.9%, *p* = 0.001, V = 0.26, moderate, 95% CI 0.206–0.463). In comparison, comorbid diabetes mellitus (68.0% vs. 39.7%, *p* = 0.010, V = 0.25, moderate) and a history of recent hospitalization (56.0% vs. 17.6%, *p* = 0.032, V = 0.39, moderate-to-large) were more common among sarcopenic individuals. BMI did not significantly differ between groups (*p* = 0.480, d = 0.35, small).

Marked impairments in physical performance were evident in sarcopenic patients. Handgrip strength was substantially lower (15.99 ± 5.39 vs. 25.27 ± 6.06 kg, *p* < 0.001, d = −1.60, very large), as were mid-arm and calf circumferences (23.12 ± 2.12 vs. 25.12 ± 2.44 cm, *p* = 0.013, d = −0.87, large; and 31.47 ± 2.41 vs. 34.76 ± 2.34 cm, *p* = 0.002, d = −1.39, very large, respectively). Sarcopenic patients had significantly lower SPPB scores (7.56 ± 2.65 vs. 10.49 ± 1.02, *p* < 0.001, d = −1.55, very large), slower gait speed (0.78 ± 0.14 vs. 0.87 ± 0.27 m/s, *p* = 0.010, d = −0.40, small), and longer TUG times (18.87 ± 2.74 vs. 15.06 ± 2.51 s, *p* < 0.001, d = 1.46, very large).

The quality-of-life assessment demonstrated that 74.4% of participants had impaired quality of life (SF-36 scores < 50). Quality of life was significantly reduced in sarcopenic individuals. SF-36 total scores were markedly lower (32.57 ± 12.03 vs. 59.45 ± 14.82, *p* = 0.001, d = −1.96, very large), and Barthel Index scores were also reduced (86.30 ± 13.69 vs. 95.85 ± 5.70, *p* = 0.010, d = −0.97, large).

Among laboratory parameters, only hemoglobin levels were significantly lower in sarcopenic patients (7.76 ± 0.97 vs. 8.49 ± 0.56 g/dL, *p* = 0.048, d = −0.96, large). No significant differences were observed for serum calcium, albumin, triglycerides, CRP, or ferritin (all *p* > 0.05; d = −0.11 to 0.32, negligible to small).

### 3.2. Sarcopenia Screening and Diagnosis

The mean SARC-F score was 2.42 ± 1.76, with significantly higher scores in sarcopenic patients (3.08 ± 0.75 vs. 1.18 ± 1.02, *p* = 0.041). Only 14 patients (11.86%) achieved SARC-F scores ≥ 4, indicating probable sarcopenia. The SARC-F demonstrated 57.6% sensitivity and 61.5% specificity in detecting sarcopenia.

Physical performance testing revealed that 33 patients (27.9%) had SPPB scores ≤ 8, indicating severe sarcopenia. Gait speed ≤ 0.8 m/s was observed in 14.6% of participants, while 21 patients (17.8%) required ≥20 s for TUG test completion.

### 3.3. Quality of Life Subscale Scores

**Figure 2:**
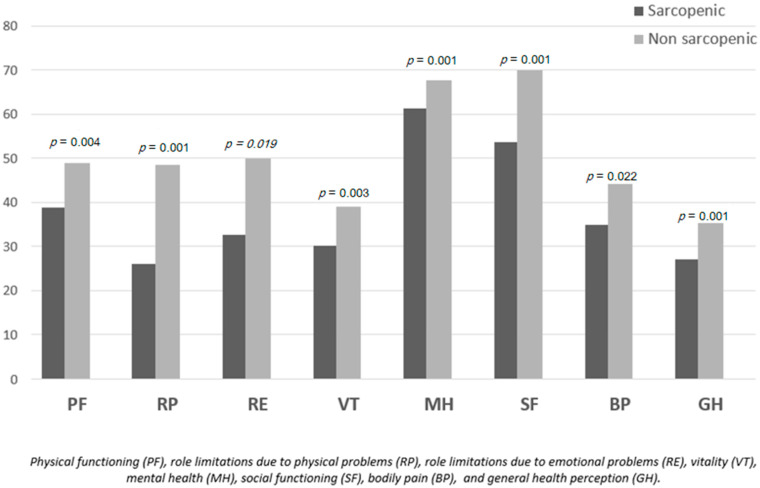
Comparison of SF-36 quality of life subscale scores between sarcopenic and non-sarcopenic patients. Sarcopenic individuals consistently reported lower scores across all domains, including physical functioning (PF), role limitations due to physical problems (RP), role limitations due to emotional problems (RE), vitality (VT), mental health (MH), social functioning (SF), bodily pain (BP), and general health perception (GH). All differences were statistically significant (*p* < 0.05).

In Figure 2, sarcopenic patients had significantly lower SF-36 scores across all domains compared to non-sarcopenic individuals. Scores were reduced in physical functioning PF (38.80 ± 20.10 vs. 48.97 ± 16.56, *p* = 0.004, d = 0.56, moderate), physical problems RP (26.00 ± 32.32 vs. 48.53 ± 35.84, *p* = 0.001, d = 0.66, moderate), and emotional problems RE (32.66 ± 39.59 vs. 49.98 ± 38.06, *p* = 0.019, d = 0.45, small to moderate). Vitality VT was also significantly lower (30.20 ± 17.20 vs. 39.04 ± 13.31, *p* = 0.003, d = 0.59, moderate). Differences were also evident in mental health MH (61.36 ± 9.75 vs. 67.71 ± 8.10, *p* = 0.001, d = 0.72, moderate to large), social functioning SF (53.70 ± 28.15 vs. 69.94 ± 24.26, *p* = 0.001, d = 0.63, moderate), bodily pain BP (34.86 ± 22.86 vs. 44.08 ± 18.99, *p* = 0.022, d = 0.45, small to moderate), and general health perception GH (27.00 ± 14.11 vs. 35.29 ± 12.54, *p* = 0.001, d = 0.63, moderate).

### 3.4. Nutritional Analysis

In Table 2, dietary assessment revealed significant differences between groups in magnesium intake (117.76 ± 76.4 vs. 212.06 ± 114.5 mg/day, *p* = 0.02, d = 0.94, large effect) and fiber consumption (14.11 ± 8.03 vs. 26.27 ± 5.65 g/day, *p* = 0.006, d = 1.80, very large effect). No significant differences were observed in energy, protein, or other micronutrient intake between groups.

### 3.5. Multivariate Analysis

In Table 3, multivariate logistic regression analysis identified three independent predictors of sarcopenia in the study population. Diabetes mellitus was associated with more than a two-fold increased risk of sarcopenia (OR = 2.14, 95% CI: 1.30–3.67, *p* < 0.001). Longer duration of hemodialysis was also a significant risk factor (OR = 1.56, 95% CI: 1.20–2.71, *p* = 0.028). Conversely, higher SPPB scores, which reflect better physical performance, were associated with a lower presence of sarcopenia (OR = 0.48, 95% CI: 0.35–0.65, *p* < 0.001); however, SPPB should be regarded as an indicator of the consequences of sarcopenia rather than an independent predictive factor.

## 4. Discussion

### 4.1. Principal Findings

This multicenter study identified a 42.4% sarcopenia prevalence among Tunisian hemodialysis patients, with diabetes mellitus, prolonged hemodialysis duration, and reduced physical performance serving as independent risk factors. Sarcopenic patients demonstrated significantly impaired quality of life and functional independence compared to non-sarcopenic individuals.

### 4.2. Sarcopenia Prevalence in Hemodialysis Populations

The observed prevalence of sarcopenia aligns with international reports, although considerable variation exists across studies. Chinese investigations reported a 59.8% prevalence [12], while British studies found 55% [16], and French research documented lower rates of 28% [16]. These variations likely reflect differences in diagnostic criteria, population characteristics, and methodological approaches.

The EWGSOP2 criteria utilized in this study represent current gold standards for sarcopenia diagnosis, emphasizing muscle strength as the primary determinant [6]. However, the observed low SARC-F sensitivity (57.6%) suggests limitations in community screening applications, consistent with previous validation studies.

### 4.3. Determining Muscle Mass in the Absence of BIA

In the absence of Bioelectrical Impedance Analysis (BIA), Dual-Energy X-ray Absorptiometry (DXA), Magnetic Resonance Imaging (MRI), and Computed Tomography (CT) are the most accurate methods for determining muscle mass; however, they are associated with higher costs and limited accessibility [31]. Anthropometric measurements offer more practical and cost-effective alternatives; however, they are less accurate and influenced by factors such as age, sex, and body fat [32].

### 4.4. Diabetes Mellitus as a Risk Factor

Diabetes mellitus emerged as the strongest independent risk factor (OR = 2.14), consistent with previous longitudinal studies. The pathophysiological relationship between diabetes and sarcopenia involves multiple mechanisms [33]. Insulin resistance, characteristic of type 2 diabetes, impairs muscle glucose uptake and protein synthesis while promoting protein degradation [34]. Chronic hyperglycemia induces oxidative stress, which damages muscle tissues and impairs mitochondrial function essential for muscle contraction [35].

Furthermore, diabetes-associated chronic inflammation, characterized by elevated pro-inflammatory cytokines (TNF-α, IL-6), activates muscle protein degradation pathways, including the ubiquitin-proteasome system. Reduced insulin-like growth factor-1 (IGF-1) levels in diabetic patients further compromise muscle regeneration and maintenance [36]. These findings support the concept that diabetes represents a systemic condition affecting multiple organ systems, with skeletal muscle serving as a primary target for metabolic dysregulation.

### 4.5. Hemodialysis Duration and Sarcopenia Development

Prolonged hemodialysis duration (OR = 1.56) represents a significant risk factor, supporting previous observations [37,38]. Extended dialysis exposure contributes to sarcopenia through multiple pathways. Chronic inflammation, perpetuated by dialysis-related factors including biocompatible membranes, catheter-related infections, and uremic toxin accumulation, promotes muscle catabolism [39].

The dialysis procedure itself induces inflammatory responses through complement activation and cytokine release, contributing to systemic inflammation [40]. Additionally, repeated protein losses during dialysis sessions, estimated at 6–8 g per session, may contribute to negative nitrogen balance and muscle wasting [41]. Uremic toxins, including indoxyl sulfate and p-cresyl sulfate, directly impair muscle function by disrupting cellular metabolism and promoting oxidative stress [42,43].

### 4.6. Physical Performance and Sarcopenia

Lower SPPB scores (OR = 0.482) were associated with sarcopenia, reflecting the functional consequences of impaired physical performance. These findings highlight the bidirectional relationship between physical performance and sarcopenia, where reduced physical activity accelerates muscle loss, while sarcopenia further compromises physical function [44].

Exercise interventions in hemodialysis patients have demonstrated beneficial effects on muscle mass, strength, and physical performance [45]. Resistance training has shown promise in attenuating muscle wasting and improving functional capacity [46].

BMI alone may not adequately reflect differences in body composition between sarcopenic and non-sarcopenic individuals (Table 1). Indeed, sarcopenia can coexist with obesity (“sarcopenic obesity”), where excess fat mass masks the loss of muscle mass, leading to similar BMI values despite markedly different functional and metabolic profiles [47]. Furthermore, in some patients—particularly those undergoing hemodialysis—fluid overload may contribute to weight stability, thereby obscuring the differences in lean mass when assessed solely by BMI [48]. This limitation of BMI as a diagnostic tool for sarcopenia is well recognized, and it has been emphasized that direct measures of body composition (e.g., DXA, BIA, or CT) are more reliable in identifying sarcopenia and sarcopenic obesity [49].

### 4.7. Quality of Life Implications

The significant quality of life impairment observed in sarcopenic patients (SF-36 scores: 32.57 vs. 59.45) underscores the clinical relevance of this condition. Sarcopenia affects multiple domains of quality of life, including physical function, social interaction, and mental health [50]. The observed functional dependence in sarcopenic patients, as measured by Barthel Index scores, further emphasizes the need for early identification and intervention [51].

Quality of life preservation represents a primary goal in hemodialysis patient management, given the substantial treatment burden and lifestyle modifications required [52]. The identification of sarcopenia as a modifiable risk factor for quality of life deterioration provides opportunities for targeted interventions [53].

### 4.8. Nutritional Considerations

The observed differences in magnesium and fiber intake between groups warrant attention. Magnesium deficiency has been associated with increased sarcopenia risk in elderly populations [54]. A negative correlation was found between magnesium consumption and sarcopenia in a cross-sectional study of 396,283 adults using the UK Biobank [55]. Magnesium serves essential roles in muscle function, including protein synthesis, energy metabolism, and muscle contraction [56,57]. Previous studies have demonstrated associations between higher dietary magnesium intake and greater muscle density in older adults [58]. Magnesium deficiency can increase the production of reactive oxygen species, leading to protein damage and contributing to muscle degradation [59]. Magnesium also acts as an antioxidant, helping to reduce oxidative stress and chronic inflammation, both of which are risk factors for sarcopenia [60]. Additionally, magnesium plays a crucial role in muscle metabolism and function through the PI3K/Akt/mTOR pathway, supporting muscle regeneration and suppressing muscle atrophy [61].

Dietary fiber intake differences may reflect variations in overall diet quality between groups. Higher fiber intake typically correlates with increased consumption of fruits, vegetables, and whole grains, which provide antioxidants and anti-inflammatory compounds that may protect against muscle wasting [62]. Recent research has highlighted the role of gut microbiota in muscle health, with fiber serving as a prebiotic that promotes the growth of beneficial bacteria [63].

### 4.9. Clinical Implications and Screening Recommendations

These findings support the implementation of routine sarcopenia screening in hemodialysis populations, particularly for patients with diabetes mellitus or prolonged treatment duration. Early identification allows for timely interventions, including nutritional counseling, exercise prescription, and medical optimization [64]. The combination of simple anthropometric measurements and functional assessments provides a practical screening approach in resource-limited settings.

Multidisciplinary care teams should incorporate sarcopenia assessment into routine clinical practice, with a particular focus on high-risk patients [19].

### 4.10. Study Limitations

Several limitations should be acknowledged. The cross-sectional design prevents the determination of a causal relationship between identified risk factors and the development of sarcopenia. Muscle mass assessment relied on anthropometric measurements rather than imaging techniques due to resource constraints, which may have compromised diagnostic accuracy. The single-center geographical focus may limit generalizability to other populations. Although we adjusted for some covariates, residual confounding cannot be excluded. Dietary intake was based on self-reported methods and is therefore subject to recall bias and reporting errors. Future longitudinal studies utilizing advanced imaging techniques would strengthen these findings.

## 5. Conclusions

Sarcopenia affects 42.4% of hemodialysis patients, with diabetes mellitus and prolonged treatment duration representing primary risk factors. The condition significantly impairs quality of life and functional independence, emphasizing the need for systematic screening and intervention programs. Early identification of high-risk patients, particularly those with diabetes or extended dialysis duration, enables the timely implementation of preventive strategies. Future research should focus on developing cost-effective screening protocols and evaluating the effectiveness of interventions in diverse hemodialysis populations.

## Figures and Tables

**Table 1:** Baseline Characteristics of Study Population.

Characteristic	Total (*n* = 118)	Sarcopenic (*n* = 50)	Non-Sarcopenic (*n* = 68)	*p*-Value	Effect Size (Cohen’s d)
Demographics					
Age (years)	56.74 ± 14.44	65.31 ± 14.19	56.15 ± 14.70	<0.001	0.632
Female sex, *n* (%)	54 (45.76)	16 (32.0)	38 (55.9)	0.001	
Hemodialysis duration (months)	99.27 ± 113.45	148.47 ± 115.20	89.53 ± 98.7	0.001	0.556
Comorbidities					-
Diabetes mellitus, *n* (%)	61 (51.7)	34 (68.0)	27 (39.7)	0.01	-
Hypertension, *n* (%)	74 (62.7)	32 (64.0)	42 (61.8)	0.32	-
Hospitalization history, *n* (%)	40 (33.9)	28 (56.0)	12 (17.6)	0.032	-
BMI (kg/m^2^)	24.41 ± 3.59	25.25 ± 4.9	23.79 ± 3.5	0.48	0.352
Physical Performance					
Handgrip strength (kg)	21.34 ± 8.18	15.99 ± 5.39	25.27 ± 6.06	<0.001	−1.604
Mid-arm circumference (cm)	24.27 ± 2.03	23.12 ± 2.12	25.12 ± 2.44	0.013	−0.866
Calf circumference (cm)	33.21 ± 2.9	31.47 ± 2.41	34.76 ± 2.34	0.002	−1.388
SPPB score	9.25 ± 2.43	7.56 ± 2.65	10.49 ± 1.02	<0.001	−1.551
Gait speed (m/s)	0.83 ± 0.23	0.78 ± 0.14	0.87 ± 0.27	0.01	−0.401
TUG test (s	16.66 ± 2.86	18.87 ± 2.74	15.06 ± 2.51	<0.001	1.460
Quality of Life					
SF-36 total score	56.89 ± 10.43	32.57 ± 12.03	59.45 ± 14.82	0.001	−1.960
Barthel Index	91.81 ± 10.93	86.30 ± 13.69	95.85 ± 5.7	0.01	−0.965
Laboratory tests					
Calcium (mmol/L)	2.53 ± 0.29	2.52 ± 0.23	2.54 ± 0.12	0.851	−0.114
Hemoglobin (g/dL)	8.12 ± 0.76	7.76 ± 0.97	8.49 ± 0.56	0.048	−0.960
Triglycerides (mmol/L)	2.36 ± 0.43	2.33 ± 0.5	2.4 ± 0.36	0.364	−0.165
Albumin (g/L)	38.87 ± 4.61	38.6 ± 4.12	39.15 ± 5.1	0.119	−0.117
CRP (mg/L)	6.88 ± 2.23	7.23 ± 2.5	6.53 ± 1.97	0.571	0.317
Ferritin (µg/L)	457.42 ± 209	484.74 ± 220	430.11 ± 198	0.293	0.263

BMI = Body mass index; SPPB = Short Physical Performance Battery; CRP = reactive protein C.

**Table 2:** Results of the dietary survey of the population.

	Total *n* = 118	Sarcopenic Group *n* = 50	Non-Sarcopenic Group *n* = 68	*p*	Effect Size (Cohen’s d)
Calories (Kcal/Kg ideal weight/day)	22.83 ± 19.54	24.56 ± 11.23	25.34 ± 13.76	0.45	0.061
Proteins (g/d)	55.54 ± 5.61	52.94 ± 4.23	57.59 ± 6.12	0.23	0.861
Lipids (g/d)	44.94 ± 7.44	40.50 ± 5.19	48.49 ± 7.45	0.1	1.21
Carbohydrates (g/d)	191.06 ± 39.1	173.44 ± 29.4	203.29 ± 42.87	0.3	0.79
Calcium (mg/d)	403.2 ± 156.21	383.29 ± 154.94	419.18 ± 156	0.211	0.231
Magnesium (mg/d)	174.51 ± 111.83	117.76 ± 76.4	212.06 ± 114.5	0.02	0.941
Vitamin D (µg/d)	2.45 ± 2.43	2.0 ± 3.8	2.78 ± 0.45	0.321	0.313
Fibers (g/d)	21.23 ± 9.67	14.11 ± 8.03	26.27 ± 5.65	0.006	1.799
Phosphorus (mg/d)	795.06 ± 266.25	747.74 ± 234	831.06 ± 291	0.128	0.310

**Table 3:** Independent predictors of sarcopenia identified by multivariate logistic regression analysis and SPPB.

Variable	Odds Ratio	IC	*p*-Value
Longer hemodialysis duration	1.56	[1.2, 2.71]	*p* = 0.028
Diabetes mellitus	2.14	[1.3, 3.67]	*p* < 0.001
SPPB high	0.482	[0.26, 0.64]	*p* < 0.001

Notes: SPPB = Short Physical Performance Battery.

## Data Availability

The data that support the findings of this study are available from the corresponding author upon reasonable request.

## References

[1] Bello A.K., Okpechi I.G., Osman M.A., Cho Y., Htay H., Jha V., Wainstein M., Johnson D.W. (2022). Epidemiology of haemodialysis outcomes. Nat. Rev. Nephrol..

[2] Petermann-Rocha F., Balntzi V., Gray S.R., Lara J., Ho F.K., Pell J.P., Celis-Morales C. (2022). Global prevalence of sarcopenia and severe sarcopenia: A systematic review and meta-analysis. J. Cachexia Sarcopenia Muscle.

[3] Cruz-Jentoft A.J., Baeyens J.P., Bauer J.M., Boirie Y., Cederholm T., Landi F., Martin F.C., Michel J.-P., Rolland Y., Schneider S.M. (2010). Sarcopenia: European consensus on definition and diagnosis: Report of the European Working Group on Sarcopenia in Older People. Age Ageing.

[4] Rosenberg I.H. (1989). Epidemiologic and methodologic problems in determining nutritional status of older persons (Summary comments). Am. J. Clin. Nutr..

[5] Morley J.E., Abbatecola A.M., Argiles J.M., Baracos V., Bauer J., Bhasin S., Cederholm T., Coats A.J.S., Cummings S.R., Evans W.J. (2011). Sarcopenia with limited mobility: An international consensus. J. Am. Med. Dir. Assoc..

[6] Carrero J.J., Stenvinkel P., Cuppari L., Ikizler T.A., Kalantar-Zadeh K., Kaysen G., Mitch W.E., Price S.R., Wanner C., Wang A.Y. (2013). Etiology of the protein-energy wasting syndrome in chronic kidney disease: A consensus statement from the International Society of Renal Nutrition and Metabolism (ISRNM). J. Ren. Nutr..

[7] Zicarelli M., Duni A., Leivaditis K., Lin Y.-L., Baciga F., Pugliese S., Fiorentino M., Hsu B.-G., Roumeliotis S., Battaglia Y. (2025). Comprehensive Insights into Sarcopenia in Dialysis Patients: Mechanisms, Assessment, and Therapeutic Approaches. Medicina.

[8] Wang X.H., Mitch W.E. (2014). Mechanisms of muscle wasting in chronic kidney disease. Nat. Rev. Nephrol..

[9] Souweine J.-S., Kuster N., Chenine L., Rodriguez A., Patrier L., Morena M., Badia E., Chalabi L., Raynal N., Ohresser I. (2018). Physical inactivity and protein energy wasting play independent roles in muscle weakness in maintenance haemodialysis patients. PLoS ONE.

[10] Workeneh B.T., Mitch W.E. (2010). Review of muscle wasting associated with chronic kidney disease. Am. J. Clin. Nutr..

[11] Slee A., McKeaveney C., Adamson G., Davenport A., Farrington K., Fouque D., Kalantar-Zadeh K., Mallett J., Maxwell A.P., Mullan R. (2020). Estimating the Prevalence of Muscle Wasting, Weakness, and Sarcopenia in Hemodialysis Patients. J. Ren. Nutr..

[12] Wu Y.-Y., Li J.-Y., Xia Q.-J., Gao Y.-Y., Zhang C., Xu P.-J., Liu J., Zhang H.-J., Yu R.-Z. (2024). Analysis of Risk Factors of Sarcopenia in Patients with Maintenance Hemodialysis and Its Correlation with Emotional Status and Quality of Life. J. Multidiscip. Healthc..

[13] Crenn P., Lévy P. (2011). Reconnaître et traiter la dénutrition en ambulatoire. Post’U FMC-HE.

[14] Abro A., Delicata L.-A., Vongsanim S., Davenport A. (2018). Differences in the prevalence of sarcopenia in peritoneal dialysis patients using hand grip strength and appendicular lean mass: Depends upon guideline definitions. Eur. J. Clin. Nutr..

[15] Smith C., Woessner M.N., Sim M., Levinger I. (2022). Sarcopenia definition: Does it really matter? Implications for resistance training. Ageing Res. Rev..

[16] Guelmami N., Aissa M.B., Ammar A., Dergaa I., Trabelsi K., Jahrami H. (2023). Guidelines for applying psychometrics in sports science: Transitioning from traditional methods to the AI Era. Tunis. J. Sports Sci. Med..

[17] Sabatino A., Cuppari L., Stenvinkel P., Lindholm B., Avesani C.M. (2021). Sarcopenia in chronic kidney disease: What have we learned so far?. J. Nephrol..

[18] Kiss C.M., Bertschi D., Beerli N., Berres M., Kressig R.W., Fischer A.M. (2024). Calf circumference as a surrogate indicator for detecting low muscle mass in hospitalized geriatric patients. Aging Clin. Exp. Res..

[19] Guralnik J.M., Simonsick E.M., Ferrucci L., Glynn R.J., Berkman L.F., Blazer D.G., Scherr P.A., Wallace R.B. (1994). A short physical performance battery assessing lower extremity function: Association with self-reported disability and prediction of mortality and nursing home admission. J. Gerontol..

[20] Peters D.M., Fritz S.L., Krotish D.E. (2013). Assessing the reliability and validity of a shorter walk test compared with the 10-Meter Walk Test for measurements of gait speed in healthy, older adults. J. Geriatr. Phys. Ther..

[21] Podsiadlo D., Richardson S. (1991). The timed “Up & Go”: A test of basic functional mobility for frail elderly persons. J. Am. Geriatr. Soc..

[22] Woo J., Leung J., Morley J.E. (2014). Validating the SARC-F: A suitable community screening tool for sarcopenia?. J. Am. Med. Dir. Assoc..

[23] Guermazi M., Allouch C., Yahia M., Huissa T., Ghorbel S., Damak J., Mrad M., Elleuch M. (2012). Translation in Arabic, adaptation and validation of the SF-36 Health Survey for use in Tunisia. Ann. Phys. Rehabil. Med..

[24] Collin C., Wade D.T., Davies S., Horne V. (1988). The Barthel ADL Index: A reliability study. Int. Disabil. Stud..

[25] Ostan R., Guidarelli G., Giampieri E., Lanzarini C., Berendsen A.A.M., Januszko O., Jennings A., Lyon N., Caumon E., Gillings R. (2018). Cross-sectional analysis of the correlation between daily nutrient intake assessed by 7-day food records and biomarkers of dietary intake among participants of the NU-AGE study. Front. Physiol..

[26] Therrien M., Byham-Gray L., Beto J. (2015). A Review of Dietary Intake Studies in Maintenance Dialysis Patients. J. Ren. Nutr..

[27] Les Références Nutritionnelles en Vitamines et Minéraux. Anses—Agence Nationale de Sécurité Sanitaire de L’alimentation, de L’environnement et du Travail 2025. https://www.anses.fr/fr/content/les-references-nutritionnelles-en-vitamines-et-mineraux.

[28] Alimentation Saine n.d. https://www.who.int/fr/news-room/fact-sheets/detail/healthy-diet.

[29] Cohen J. (1988). Statistical Power Analysis for the Behavioral Sciences.

[30] Kim H.-Y. (2017). Statistical notes for clinical researchers: Chi-squared test and Fisher’s exact test. Restor. Dent. Endod..

[31] Sergi G., De Rui M., Stubbs B., Veronese N., Manzato E. (2017). Measurement of lean body mass using bioelectrical impedance analysis: A consideration of the pros and cons. Aging Clin. Exp. Res..

[32] Duarte C.K., De Abreu Silva L. (2025). What is the value of anthropometry for estimating muscle mass?. Curr. Opin. Clin. Nutr. Metab. Care.

[33] Souweine J.-S., Pasquier G., Morena M., Patrier L., Rodriguez A., Raynal N., Ohresser I., Benomar R., Hayot M., Mercier J. (2024). Beyond sarcopenia: Frailty in chronic haemodialysis patients. Clin. Kidney J..

[34] Souza A.B.F., Nascimento D.A.C., Rodrigues I.J.M., Charone C.C.O., Lopes G.L., Lima R.S., Sá A.A., Carneiro T.X., Moraes N.S. (2019). Association between sarcopenia and diabetes in community dwelling elderly in the Amazon region—Viver Mais Project. Arch. Gerontol. Geriatr..

[35] Sugimoto K., Wang C.-C., Rakugi H. (2016). Sarcopenia in diabetes mellitus. Musculoskeletal Disease Associated with Diabetes Mellitus.

[36] Chen H., Huang X., Dong M., Wen S., Zhou L., Yuan X. (2023). The Association Between Sarcopenia and Diabetes: From Pathophysiology Mechanism to Therapeutic Strategy. Diabetes Metab. Syndr. Obes..

[37] Purnamasari D., Tetrasiwi E.N., Kartiko G.J., Astrella C., Husam K., Laksmi P.W. (2022). Sarcopenia and Chronic Complications of Type 2 Diabetes Mellitus. Rev. Diabet. Stud..

[38] Zhao Q., Zhu Y., Zhao X., Shi R., Lu T., Yu R., Wang D. (2024). Prevalence and risk factors of sarcopenia in patients on maintenance hemodialysis: A retrospective cohort study. BMC Musculoskelet. Disord..

[39] Ozen A., Cakmak E., Bilen N., Akel M., Gurel A., Selcuk M. (2021). Investigation of sarcopenia in hemodialysis patients in Adiyaman province. Med.-Sci..

[40] Noce A., Marrone G., Ottaviani E., Guerriero C., Di Daniele F., Pietroboni Zaitseva A., Di Daniele N. (2021). Uremic Sarcopenia and Its Possible Nutritional Approach. Nutrients.

[41] Kim J.C., Kalantar-Zadeh K., Kopple J.D. (2013). Frailty and Protein-Energy Wasting in Elderly Patients with End Stage Kidney Disease. J. Am. Soc. Nephrol..

[42] Ikizler T.A., Flakoll P.J., Parker R.A., Hakim R.M. (1994). Amino acid and albumin losses during hemodialysis. Kidney Int..

[43] Vanholder R., Schepers E., Pletinck A., Nagler E.V., Glorieux G. (2014). The uremic toxicity of indoxyl sulfate and p-cresyl sulfate: A systematic review. J. Am. Soc. Nephrol..

[44] Hu H., Chau P.H., Choi E.P.H. (2024). Physical activity, exercise habits and health-related quality of life in maintenance hemodialysis patients: A multicenter cross-sectional study. J. Nephrol..

[45] Olvera-Soto M.G., Valdez-Ortiz R., Alvarenga J.C.L., Espinosa-Cuevas M.Á. (2016). Effect of resistance exercises on the indicators of muscle reserves and handgrip strength in adult patients on hemodialysis. J. Ren. Nutr..

[46] Chung Y.-C., Yeh M.-L., Liu Y.-M. (2017). Effects of intradialytic exercise on the physical function, depression and quality of life for haemodialysis patients: A systematic review and meta-analysis of randomised controlled trials. J. Clin. Nurs..

[47] Prado C.M.M., Wells J.C.K., Smith S.R., Stephan B.C.M., Siervo M. (2012). Sarcopenic obesity: A Critical appraisal of the current evidence. Clin. Nutr..

[48] Mori K. (2021). Maintenance of Skeletal Muscle to Counteract Sarcopenia in Patients with Advanced Chronic Kidney Disease and Especially Those Undergoing Hemodialysis. Nutrients.

[49] Cruz-Jentoft A.J., Bahat G., Bauer J., Boirie Y., Bruyère O., Cederholm T., Cooper C., Landi F., Rolland Y., Sayer A.A. (2019). Sarcopenia: Revised European consensus on definition and diagnosis. Age Ageing.

[50] Giglio J., Kamimura M.A., Lamarca F., Rodrigues J., Santin F., Avesani C.M. (2018). Association of Sarcopenia With Nutritional Parameters, Quality of Life, Hospitalization, and Mortality Rates of Elderly Patients on Hemodialysis. J. Ren. Nutr..

[51] Gould D.W., Watson E.L., Wilkinson T.J., Wormleighton J., Xenophontos S., Viana J.L., Smith A.C. (2019). Ultrasound assessment of muscle mass in response to exercise training in chronic kidney disease: A comparison with MRI. J. Cachexia Sarcopenia Muscle.

[52] Ibrahim N., Teo S.S.L., Che Din N., Abdul Gafor A.H., Ismail R. (2015). The Role of Personality and Social Support in Health-Related Quality of Life in Chronic Kidney Disease Patients. PLoS ONE.

[53] Spiegel B.M.R., Melmed G., Robbins S., Esrailian E. (2008). Biomarkers and health-related quality of life in end-stage renal disease: A systematic review. Clin. J. Am. Soc. Nephrol..

[54] Barbagallo M., Veronese N., Dominguez L.J. (2021). Magnesium in Aging, Health and Diseases. Nutrients.

[55] Petermann-Rocha F., Chen M., Gray S.R., Ho F.K., Pell J.P., Celis-Morales C. (2020). Factors associated with sarcopenia: A cross-sectional analysis using UK Biobank. Maturitas.

[56] Castiglioni S., Cazzaniga A., Albisetti W., Maier J.A.M. (2013). Magnesium and osteoporosis: Current state of knowledge and future research directions. Nutrients.

[57] Ganapathy A., Nieves J.W. (2020). Nutrition and Sarcopenia—What Do We Know?. Nutrients.

[58] Rude R.K., Singer F.R., Gruber H.E. (2009). Skeletal and hormonal effects of magnesium deficiency. J. Am. Coll. Nutr..

[59] Yang S.-W., Chen Y.-Y., Chen W.-L. (2022). Association between oral intake magnesium and sarcopenia: A cross-sectional study. BMC Geriatr..

[60] Suranto A., Hermina S., Dwi N., Lusiana B. (2020). Correlation between serum magnesium level and sarcopenia occurence in the elderly women: Study with dual-energy x-ray absorptiometry (DXA). Malays. J. Med. Health Sci..

[61] Cui C., Bao Z., Chow S.K.-H., Wong R.M.Y., Welch A., Qin L., Cheung W.H. (2022). Coapplication of Magnesium Supplementation and Vibration Modulate Macrophage Polarization to Attenuate Sarcopenic Muscle Atrophy through PI3K/Akt/mTOR Signaling Pathway. Int. J. Mol. Sci..

[62] Hanna R.M., Ghobry L., Wassef O., Rhee C.M., Kalantar-Zadeh K. (2020). A Practical Approach to Nutrition, Protein-Energy Wasting, Sarcopenia, and Cachexia in Patients with Chronic Kidney Disease. Blood Purif..

[63] Zhou Q., Zhang H., Yin L., Li G., Liang W., Chen G. (2022). Characterization of the gut microbiota in hemodialysis patients with sarcopenia. Int. Urol. Nephrol..

[64] Cai G., Ying J., Pan M., Lang X., Yu W., Zhang Q. (2022). Development of a risk prediction nomogram for sarcopenia in hemodialysis patients. BMC Nephrol..

[65] Dergaa I., Ben Saad H., Glenn J.M., Ben Aissa M.M., Taheri M., Swed S., Guelmami N., Chamari K. (2024). A thorough examination of ChatGPT-3.5 potential applications in medical writing: A preliminary study. Medicine.

[66] Dergaa I., Fekih-Romdhane F., Glenn J.M., Saifeddin Fessi M., Chamari K., Dhahbi W., Zghibi M., Bragazzi N.L., Ben Aissa M., Guelmami N. (2023). Moving beyond the stigma: Understanding and overcoming the resistance to the acceptance and adoption of artificial intelligence chatbots. New Asian J. Med..

